# A Simple Synthesis and Microstructure Analysis of Human Peptide LL-37@Gold Nanoparticles (Known as LL-37@AuNPs) Conjugates as Antimicrobials and Substances for Wound Healing

**DOI:** 10.3390/ma16247675

**Published:** 2023-12-16

**Authors:** Subaer Subaer, Hartati Hartati, Imam Ramadhan, Harlyenda Ismayanti, Agung Setiawan

**Affiliations:** 1 Physics Department, Faculty of Mathematics and Natural Science, Universitas Negeri Makassar, Makassar 90222, Indonesia; ir221299@gmail.com (I.R.); harlyendaismayanti@gmail.com (H.I.); 2 Green of Excellence of Green Materials & Technology (CeoGM-Tech) FMIPA, Universitas Negeri Makassar, Makassar 90222, Indonesia; hartati@unm.ac.id; 3 Biology Department, Faculty of Mathematics and Natural Science, Universitas Negeri Makassar, Makassar 90222, Indonesia; 4 Research Center for Mining Technology, National Research and Innovation Agency (BRIN), Building 820, KST B.J. Habibie, Banten 15314, Indonesia; agung.metallurgy@gmail.com

**Keywords:** AuNPs, conjugate, LL-37 antimicrobial peptide, microstructure, wound healing

## Abstract

The basis of the present study is a straightforward method involving fewer chemical species for conjugating gold nanoparticles (AuNPs) with the antimicrobial peptide LL-37 designated as LL-37@AuNPs. Investigating the microstructure characteristics of the resulting materials and their potential as antibacterial and wound-healing substances are the main objectives of this study. Zeta (ζ) potential, Fourier transform infrared (FTIR), X-ray diffraction (XRD), field effect scanning electron microscopy (FE-SEM), energy dispersive X-ray diffraction (EDS), transmission electron microscopy (TEM), and UV-Vis spectrophotometry were used to analyze the physico–chemical properties of LL-37@AuNPs. The magnitude of LL-37′s zeta potential and the LL-37@AuNPs show that the specimens are electrically stable and resistant to flocculation and coagulation. The surface plasmon resonance (RPS) of AuNPs, which is positioned at a wavelength of about 531 nm, was found to be unaffected by the presence of the LL-37 antimicrobial peptide. The FTIR data show the functional group characteristics of the LL-37@AuNPs vibration bands, and the XRD diffractogram confirms the formation of the LL-37@AuNPs conjugate nanocomposite. Based on FE-SEM and TEM data, the bulk of AuNPs were found to have a circular shape, with an average size of about 22.88 ± 8.21 nm. It was discovered that the LL-37@AuNPs had a good ability to inhibit *S. aureus* from growing. The wound-healing percentage reached 85% on day 12 of the trial, significantly greater than the results of the negative controls. LL-37@AuNPs(4) is the sample that had the highest percentage of wound healing between days 3 and 12. Moreover, sample LL-37@AuNPs(4) contains 0.45 µL of LL-37, whereas sample LL-37@AuNPs(2) contains 0.22 µL of LL-37. The faster wound-healing rate in LL-37@AuNPs(4) was believed to be due to a higher concentration of LL-37, which was able to stop *S. aureus* from developing while suppressing the inflammation surrounding the wound. The study’s findings reveal that LL-37@AuNPs might be made using a straightforward process, making them a powerful antibacterial and therapeutic substance. However, before this discovery is applied in the field of medicine, a more thorough investigation is necessary.

## 1. Introduction

Nanoparticles (NPs), or materials with dimensions that range between 1 and 100 nm, offer numerous applications in the industrial, medicinal, and technical sectors. NPs have the potential to be exploited as therapeutic materials or as carriers of drugs due to their unique physical and chemical properties. Metals and natural or synthetic polymers can all be used to create NPs. NPs help in reducing side effects by lengthening the period of a drug’s stay in the body through effectively delivering drugs to the designated location.

The usage of such a drug-delivery method also shields the medication from the body’s rapid excretion and condition of breakdown. These nanoparticles are appealing for medical applications because of their distinctive characteristics, which include their far higher surface-to-mass ratio, and their capacity to adsorb and transfer other substances. NPs such as AuNPs and AgNPs are able to bind, adsorb, and transport various molecules, including medications, plant extracts, and peptides, because of their comparatively broad functional surface [1,2,3].

The development of novel antibiotics to combat bacterial resistance has been the focus of numerous studies carried out in recent years. Antimicrobial peptide (AMP) structure and function have become hot topics in research. Because they perform direct antibacterial and mediator functions and operate as the host’s first line of defense against invasive pathogens, AMPs are an essential component of the innate immune system found in the majority of living things. The cationic antimicrobial peptide LL-37, which has been demonstrated to be effective against a variety of bacterial agents, is the sole member of the human cathelicidin, also known as hCAP18 (human cationic antimicrobial peptide), which was discovered in 1995 [4,5].

Proprotein hCAP18, an inactive version of cathelicidin, is the source of cathelicidin in humans. When processing occurs, the active C-end or terminal of 37 amino acids (LLGDFFRKSKEKIGKEFKRIVQRIKDFLRNLVPRTES) is released. The cathelicidin gene encodes an 18 kDa hCap-18 protein precursor. The first two residues of leucine’s 37 amino acid residues make up the C-terminal end, known by its scientific name LL-37. The primary source of LL-37 is polymorphonuclear leucocytes, although monocytes and epithelial cells are also capable of producing it [6]. With 11 bases and 5 acids, 54% of cathelicidin residues are hydrophilic, meaning that at physiological pH, these molecules possess a positive charge of ±6. Helicidin has a circular dichroism spectrum in an aqueous solution, which is compatible with its irregular structure. In lipophilic environments, on the other hand, many amino acids are able to generate intramolecular hydrogen bonds in membranes, which lock the secondary structure into an a-helix [7]. 

The wide range of antimicrobial activity of AMPs against fungi, bacteria, and protozoa, with minimum inhibitory concentrations, has generated interest [8,9,10]. It has been demonstrated that a few cationic peptides can stop the spread of enveloped viruses, including the human immunodeficiency virus [5] and cancer development [11]. These peptides may also be able to overcome bacterial resistance, inhibit and destroy bacterial biofilms [9,12,13], wound healing [14], and human gingival fibroblasts [15], and the possibility of preventing heat stress-induced intestinal damage, inflammatory response, and multiple organ dysfunction [16]. As a result, AMPs or their derivatives may offer a novel class of antimicrobial medications. 

The immobilization of AMPs in NPs is one method to distribute AMPs in vivo, shield them from potential toxicity, and strengthen their resistance to protease breakdown. Comparing free AMPs to AMPs–NPs conjugate, the latter may provide reduced toxicity, increased antibacterial activity, superior targeting, and higher stability [1,10,17,18]. According to Comune et al. [10], LL37-AuNPs can be conjugated using a reasonably straightforward one-step technique, which enables the manufacture of these compounds on a broad scale at a relatively inexpensive cost. Further investigation on the potential of conjugating AMPs and AuNPs for wound-healing therapy has been conducted, and the results were very encouraging [14,16]. 

It appears that the qualities of a mixture of two or more elements are superior to those of the separate materials. The majority of composite materials are made up of two phases: a continuous matrix that serves as a binder and a discontinuous reinforcing element. Applications for nanocomposite materials have been growing at a rapid pace. These days, anticorrosive barrier protection, drug delivery, engineering, and UV-protecting gels all use these nanocomposites. In contrast to other research that has been published, the current work used a comparatively simple technique that involved fewer chemical species to conjugate the LL-37 antimicrobial peptide with gold nanoparticles (AuNPs). The next step is characterizing the conjugate materials of LL-37@AuNPPs in terms of their physico–chemical characteristics, antibacterial resistance, and wound-healing abilities.

## 2. Materials and Methods

### 2.1. Synthesis of AuNPs

AuNPs were synthesized using the Turkevich method described by Dong et al. [19], which involves reducing gold (III) chloride trihydrate (HAuCl_4_⋅3H_2_O) with trisodium citrate (Na_3_C_6_H_5_O_7_), both materials supplied by Sigma-Aldrich, St. Louis, MO, USA. A magnetic stirrer was used to stir 1 mM of chloride trihydrate at 1000 rpm after it had been dissolved in 100 mL of distilled water. The solution was brought to a boil by increasing its temperature. After that, 5 mL of 1 wt% trisodium citrate was added to the solution and shaken for three minutes, or until the solution turned red-wine in color, signifying the reduction of Au(ii) to Au°. Following a 25-min centrifugation at 1000 rpm, the solution was allowed to cool to ambient temperature. The solution containing AuNPs was kept cold, at 4 °C, for further examination. The resulting AuNPs surface plasmon resonance (SPR) was investigated using a UV-Vis spectrophotometer (UV-Vis). Fourier transform infrared (FTIR) was used to examine the functional groups of AuNPs. The morphology of AuNPs at the nanoscale level was examined using transmission electron microscopy (TEM) (FEI Company, Eindhoven, The Netherlands), and X-ray diffraction (XRD) was used to examine the crystalline structure of AuNPs.

### 2.2. Synthesis of LL-37@AuNPs Conjugates

The LL-37 antimicrobial peptide (C_205_H_340_N_60_O_53_) with 95.3% purity (HPLC), which weighed up to 15 mg, was acquired from Elabscience^®^ (USA) via CV Gamma Science Biolab Indonesia and utilized exactly as supplied. For this study, we produced LL-37 antimicrobial peptide in quantities of 1 mg (0.22 µmol) and 2 mg (0.45 µmol), homogeneously diluted in demineralized water, and mixed it with 1 mL of the as-prepared AuNPs solution. The specimens were identified as LL-37@AuNPs(2) and LL-37@AuNPs(4). Following two hours of cooling at 4 °C, the mixture was centrifuged for 5 min at 250 rpm. Then, zeta potential measurement, UV-Vis, FTIR, XRD, FE-SEM, TEM, antibacterial measurement, and wound-healing studies were performed on the produced specimens.

#### 2.2.1. Zeta Potential

Zeta potential was estimated using a HORIBA Scientific SZ-100 particle size analyzer (PSA) (HORIBA, Ltd., Kyoto, Japan) and the software of HORIBA SZ-100 for Windows (Z Type) ver. 2.50 was provided by the manufacturer. For this purpose, 2 mL of each sample was measured in a disposable cuvette. The samples were measured three times at a temperature of 25 °C with an electrode voltage of 3.9 V.

#### 2.2.2. UV-Vis Measurement

Ultraviolet–visible absorbance spectra of AuNPs and PEP-AuNPs were acquired using a Shimadzu UV-1280 multipurpose UV-Vis spectrophotometer (Kyoto, Japan). AuNPs and LL-37@AuNPs specimens were deposited in a 96-well collection plate at a volume of 100 μL. All spectra were recorded at a resolution of 1 nm over a wavelength range of 300–800 nm.

#### 2.2.3. Fourier Transform Infrared (FTIR) Measurement

Infrared (IR) spectra measurements of AuNPs and LL-37@AuNPs were conducted using a Shimadzu, IR Prestige 21 FTIR. Liquid specimens were deposited onto disposable PTFE-IR sample cards (5 cm × 10 cm) (Sigma-Aldrich Z422797, St. Louis, MO, USA) and dried under a vacuum overnight. All spectra were recorded at a resolution of 1 cm^−1^ over a wavenumber range of 400–4000 cm^−1^. 

#### 2.2.4. X-ray Diffraction (XRD) Measurement

The diffractogram of AuNPs and LL-37@AuNPs conjugates was measured using a Rigaku MiniFlex II X-ray diffraction (XRD) (Rigaku, Tokyo, Japan) unit operating at 30 kV and 15 mA at a scanning rate of 2°/min from 10° to 90° 2θ. The XRD patterns were analyzed using the powder diffraction analysis software HighScore Plus ver. 5.2.0. The liquid samples were deposited on the XRD glass holder and vacuum-dried overnight. 

#### 2.2.5. Field-Effect Scanning Electron Microscopy (FE-SEM) Measurement

The morphology of AuNPs and LL-37@AuNPs was examined by using field-emission scanning electron microscopy (FE-SEM) (FESEM Thermo Scientific Quattro S completed with EDS detector (Waltham, MA, USA), operating at 30 kV and magnification up to 2,000,000×). The liquid specimens of AuNPs and LL-37@AuNPs were dropped onto a 10 mm × 10 mm corning glass and vacuum-dried prior to SEM examination.

#### 2.2.6. Transmission Electron Microscopy (TEM) Measurement

FEI Tecnai G2 20S-Twin electron microscope (FEI Company, Eindhoven, The Netherlands) was utilized to obtain TEM images of AuNPs and LL-37@AuNPs. The microscope featured a 200 kV acceleration voltage, 0.14 nm line resolution, and a magnification of up to 1030 kx. As much as 5 µL of the specimen suspension was dropped onto a copper-coated TEM grid, and the samples were allowed to dry at room temperature before being subjected to TEM imaging.

### 2.3. Antibacterial Measurement

The agar diffusion method was used to assess the inhibitory capabilities of LL-37@AuNPs conjugate against micro-organism formation. *Staphylococcus aureus*, *Escherichia coli*, *Bacillus cereus*, and *Candida albicans* were employed as microbe specimens. The positive control was 100 g/mL of chloramphenicol, and the negative control was distilled water. The nutrient agar (NA) medium was heated until completely dissolved, then cooled to 40 °C. A swab was used to extend the suspension of bacteria specimens. A negative control and a positive control were inserted onto a paper disc in 50 µL test specimens. All discs were incubated for 24 h at 37 °C. Following that, the diameter of the clear zone generated around the paper disc was measured, and the procedure was repeated three times. Using calipers, the inhibition or clear zone was examined and quantified, and an average of four measurements was made, which is stated mathematically as:

(1) $$\mathrm{Diameter}\;\mathrm{of}\;\mathrm{inhibition}\;\mathrm{zone}\;x=\frac{a+b+c+d}{4}$$

### 2.4. Wound-Healing Method

A healthy albino male for the in vivo investigation, and Wistar rats weighing 200–250 g were chosen. Prior to receiving the medication, the test animals at the UNM Biology Laboratory were allowed unrestricted access to food and water and required acclimatization for seven days. 120 mg/kg of ketamine was administered subcutaneously to the rats to induce anesthesia. The ethical committee for animal research at Hasanuddin University in Indonesia’s College of Medicine accepted the study with a permission code No. 0058/KKEH/RSHUH/EC/2023. 

After shaving each rat’s dorso-costal region, a 6 mm sharp skin biopsy punch was used to create the excision wound. Four groups, each with six animals, were randomly selected from among the animals. As a negative control, Group 1 was exposed to 0.5 g of ointment base; as a positive control, Group 2 received 5% povidone-iodine; Group 3 received the sample LL-37@AuNPs(2) conjugate; and Group 4 received the sample LL-37@AuNPs(4) conjugate. The test animals’ skin injuries were promptly healed with the application of the treatment. For 12 days, the treatment was administered every 24 h. Every animal wound had an identical procedure during the trial. Following injury treatment (day 0), the wound size area was assessed. On days 3, 6, 9, and 12, further wound measurements were performed. The following Equation (2) was used to determine the percentage of wound closure.

(2) $$\mathrm{Wound}\;\mathrm{closure}=\frac{\left(initial\;wound\;size-specific\;day\;wound\;size\right)}{\left(Initial\;wound\;size\;\right)}\times 100\%$$

## 3. Results and Discussion

### 3.1. Zeta Potential (ζ)

**Figure 1:**
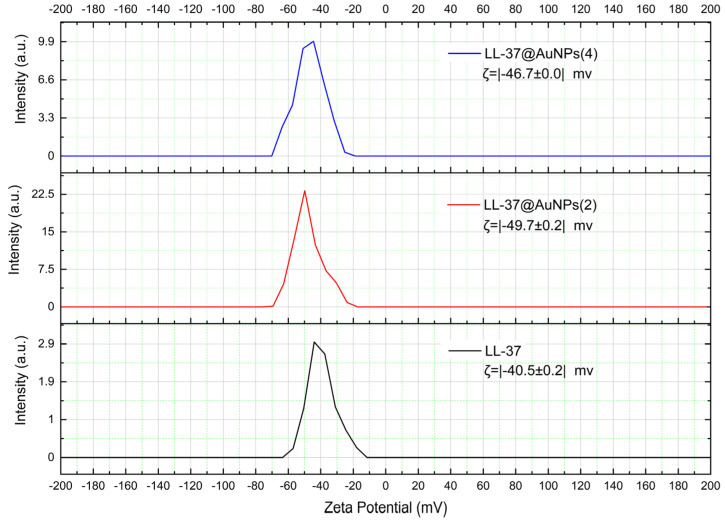
The graph and the average magnitude of zeta potential for sample LL-37 antimicrobial peptide, LL-37@AuNPs(2), and LL-37@AuNPs(4) conjugates.

Zeta potential (ζ) is one of the main forces bridging particles’ interactions. The magnitude of the zeta potential indicates the degree of electrostatic repulsion between similar particles in a dispersion. For very small molecules or particles, large zeta potential plays a role in preventing aggregation. Therefore, colloids with high zeta potential (negative or positive) are electrically stable, while colloids with low zeta potential tend to coagulate or flocculate. Figure 1 shows the typical of zeta potential of sample LL-37 antimicrobial peptide, LL-37@AuNPs(2) and LL-37@AuNPs(4).

The zeta potential of LL-37 AMPs increases significantly when they are linked to AuNPs, as Figure 1 illustrates. The samples’ respective zeta potentials vary from $\left|-40.5\pm 0.2\right|\;mV$ for LL-37 AMPs to $\left|-49.7\pm 0.2\right|\;mV$ for LL-37@AuNPs(2). The magnitudes of these zeta potentials are in good accordance with the findings of Comune et al. [10], who observed that LL37-AuNPs in the HEPES buffer had a negative zeta potential of $\left|-43.7\pm 2.9\right|\;mV$. According to these findings, the LL-37@AuNPs used in this study are resistant to coagulation and flocculation and are electrically stable.

### 3.2. UV-Vis and FTIR Measurements

Shimadzu UV-1280 multipurpose UV-Vis spectrophotometers were used to quantify the surface plasmon resonance (SPR) of AuNPs and LL-37@AuNPs. The resonant oscillation of conduction electrons at the interface of materials with positive and negative permittivity caused by incoming light is known as surface photo resonance (SPR). SPR is the foundation of several standard methods for evaluating substance adsorption onto planar metal surfaces (often gold or silver) or the surfaces of metal nanoparticles. This idea underpins a wide range of color-based biosensor applications, lab-on-a-chip sensors, and diatom photosynthesis.

**Figure 2:**
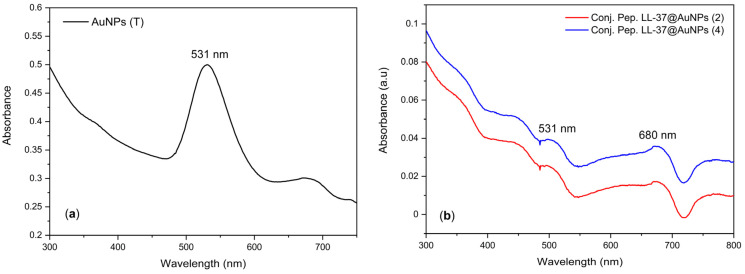
UV-Vis spectrum of (**a**) AuNPs and (**b**) LL-37@AuNPs conjugates.

Figure 2a,b show that the SPR peaks of AuNPs are located around 531 nm. The presence of LL-37 in LL-37@AuNPs conjugation does not alter the position of the SPR peaks of AuNPs, although strong UV absorption occurs around 680 nm. These results indicate that LL-37@AuNPs conjugate is highly photo-stable. This outcome and the one published by Comune et al. [10] and Xu et al. [14] are fairly consistent. The researchers produced nanorods gold (AuNRs) and pointed out that there are simultaneous transversal and longitudinal oscillations on the surface of gold nanorods because of the anisotropy of their size. Since the longitudinal plasmon peak’s position and the aspect ratio of the AuNRs are linearly related, it is possible to artificially modify it to suit specific requirements. AuNRs have this advantage over gold nanospheres as well. The different shapes and sizes of the AuNPs produced in this study are displayed in the TEM images. However, it was discovered that the size or shape of AuNPs and LL-37@AuNP conjugates had little impact on their photostability.

**Figure 3:**
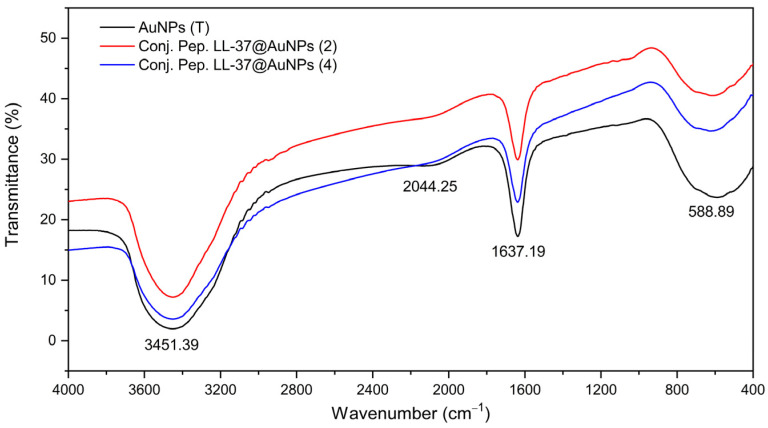
FTIR of AuNPs and LL-37@AuNPs conjugates.

Shimadzu IR Prestige 21 FTIR was employed to detect the involvement of different functional groups present in a solution of HAuCl_4_.3H_2_O and Na_3_C_6_H_5_O_7_ of the gold solution and LL-37@AuNPs conjugates shown in Figure 3. A broader peak observed at 3451.39 cm^−1^ is attributed to the O–H stretch. Two more peaks were found at 1637.19 cm^−1^ and 2044.25 cm^−1^, corresponding to the presence of (C–C stretch, alkene), and (C–C stretch, alkyne), respectively. The absorption band at 588.89 cm^−1^ is identified as (C–Cl stretch, alkyl halide). The presence of alkenes may be responsible for the reduction of gold (III) ions to AuNPs [20].

### 3.3. X-ray Diffraction Characterization (XRD)

**Figure 4:**
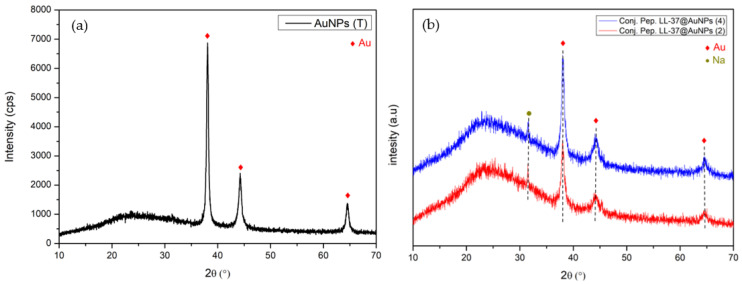
Diffractogram of (**a**) AuNPs, and (**b**) LL-37@AuNPs(2) and LL-37@AuNPs(4) showing the formation of nanocomposites.

Figure 4a reveals the diffractogram of AuNPs, which displays the peaks corresponding to the face-centered cubic (FCC) structure of the Bragg’s reflections in the planes of (111) (38.12°), (200) (44.28°), (220) (64.72°), (311) (77.78°), and (222) (82.06°). This finding implies that the synthesized AuNPs’ purity is outstanding and is consistent with the ICDD#01-071-4614 database. By applying the Debye–Scherrer formula, the average crystallinity size of AuNPs was determined to be approximately 20 nm. The diffractogram of specimens LL-37@AuNPs(2) and LL-37@AuNPs(4) is presented in Figure 4b. Due to LL-37 AMP’s low intensity and XRD amorphous nature, the combination of AuNPs and LL-37 AMPs does not appear to have significantly altered the AuNPs’ diffraction pattern, with the exception of the broad hump that formed at around 25° 2θ.

### 3.4. Field Emission-Scanning Electron Microscopy (FE-SEM)

**Figure 5:**
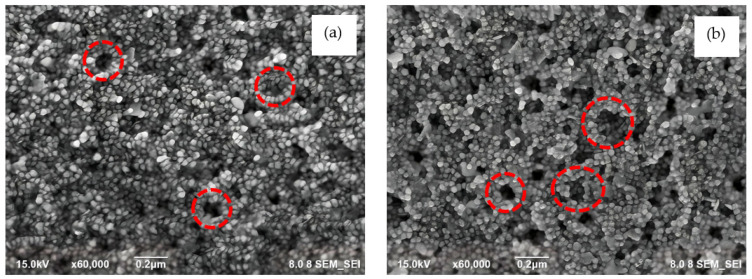
FE-SEM images of (**a**) LL-37@AuNPs(2) and (**b**) LL-37@AuNPs(4) conjugates showing the round shaped of AuNPs and the formation of cluster-like of LL-37@AuNPs conjugates (showing in circles).

The FE-SEM images of LL-37@AuNPS(2) and LL-37@AuNPs(4) in Figure 5a,b indicate the production of well-structured nanocomposites. On corning glass, the specimen for FE-SEM measurements was prepared by repeatedly dropping the LL-37@AuNPs solution and then vacuum-drying it. The images unambiguously show how the LL-37 peptide material forms a cluster-like interaction with AuNPs in a round shape. The enormous amount of specimen solution used in their production could be the cause of clusters like LL-37@AuNPs (shown in circle lines). Furthermore, it is noticeable that the LL-37 antimicrobial peptide’s polymeric characteristics and the coulombic attraction between the Au ion and the peptide’s net positive charge contribute to the relatively strong physical bonding between AuNPs and the peptide. This mechanism is possible because LL-37 peptide has a thiol group at C-terminus, which interacts with the surface of AuNPs through Au–thiol interactions [10].

**Figure 6:**
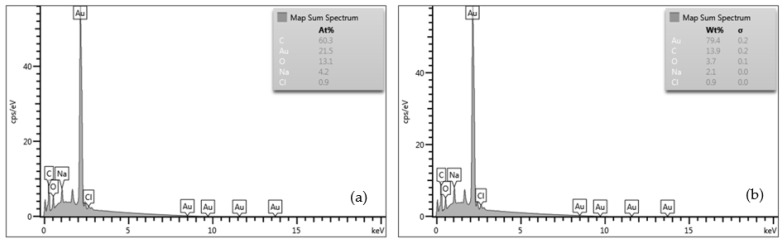
(**a**) EDS results of LL-37@AuNPs(2), and (**b**) LL-37@AuNPs(4), showing the atomic % of the chemical compositions of the specimens.

The microanalysis in terms of the atomic percentage (%) of the LL-37@AuNPs(2) and LL-37@AuNPs(4) was performed on the surface of the samples based on the energy dispersive X-ray spectroscopy (EDS) by using back-scattered electron is shown in Figure 6a,b.

The EDS results presented in Figure 6a,b clearly display the distribution of Au, C, O, Na, and Cl on the surface of the specimens originating from raw materials used to produce the conjugation materials. The presence of Na and Cl originated from chloride trihydrate (HAuCl_4_⋅3H_2_O) and trisodium citrate (Na_3_C_6_H_5_O_7_) solution used in the synthesis of AuNPs.

### 3.5. Transmission Electron Microscopy (TEM)

**Figure 7:**
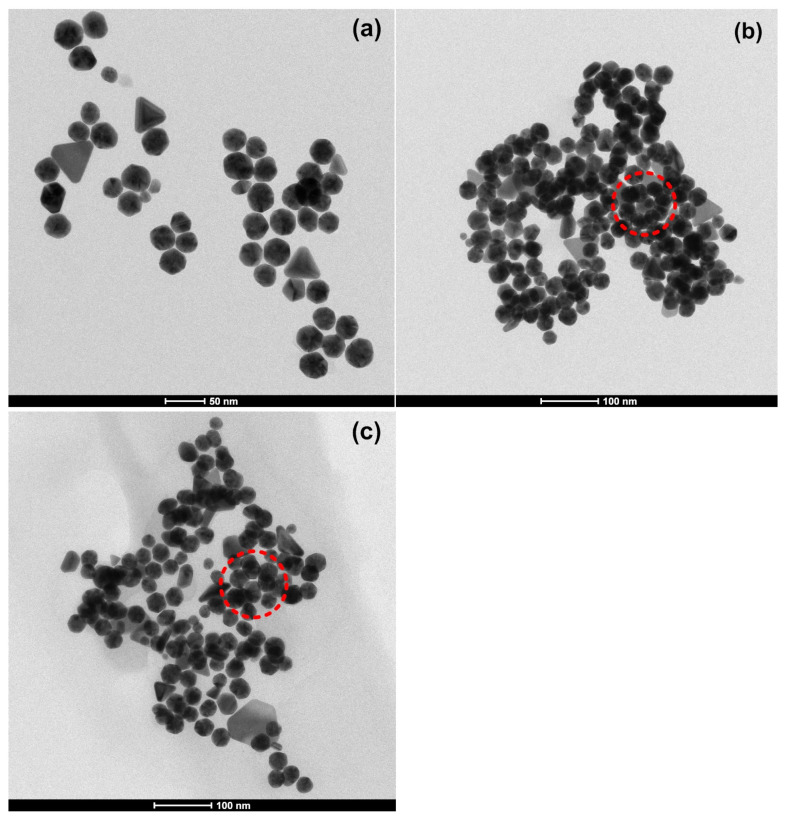
TEM images of (**a**) AuNPs, (**b**) LL-37@AuNPs(2), and (**c**) LL-37@AuNPs(4).

The TEM image of AuNPs shown in Figure 7 indicates that spherical, cylindrical, triangular, pentagonal, and hexagonal-shaped gold nanoparticles were formed with an average particle diameter of $\left|22.88\pm 8.21\right|\;nm$. The average size of AuNPs based on TEM measurement is in good agreement with the XRD results. The gold nanoparticles were well dispersed in the reaction medium without any visible agglomeration. The shape and the size of AuNPs produced in this study are in good agreement with those reported by others [19,20,21,22].

Similar TEM morphologies of LL-37@AuNPs were observed by Casciaro et al. [17] and Comune et al. [10]. The great mobility of AuNPs’ nanomaterial size enables peptides to reach their targets fast and cause the bacteria to break down and leak their internal components out through breaches in their cell membranes. TEM images also exhibit clusters resembling LL-37@AuNPs (circled lines) similar to those shown in FE-SEM images.

### 3.6. Antibacterial Properties of LL-37@AuNPs Conjugate

**Figure 8:**
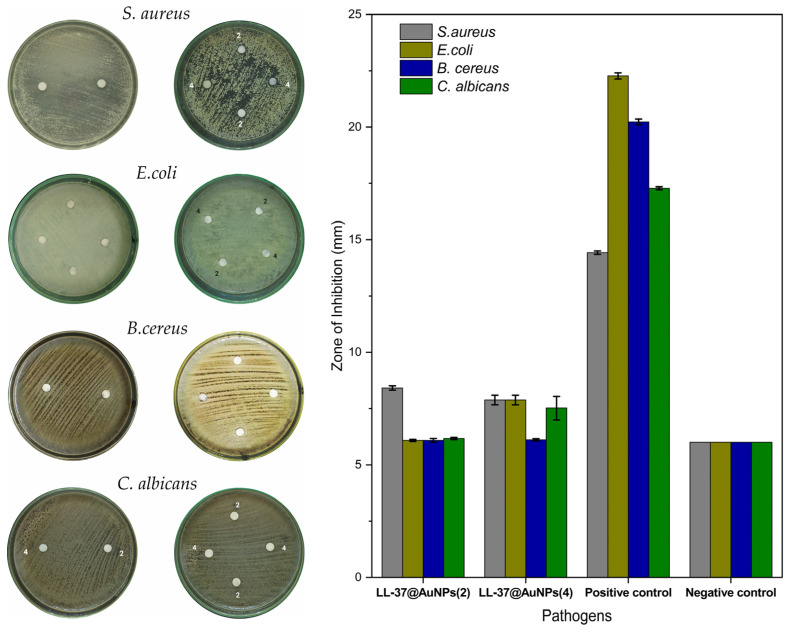
Antimicrobial assay by diffusion method.

The ability of LL-37@AuNPs as antimicrobial is observed by the development of a clear zone or inhibit zone around the paper disc. Figure 8 shows the results of antibacterial testing for the LL-37@AuNPs conjugates was performed on two samples with different concentrations of peptide LL-37.

In the test of antimicrobial activity of *S. aureus*, *E. coli*, *B. cereus*, and *C. albicans* in Figure 8 using a positive control, namely, Chloramphenicol, and a negative control, namely, Nystatin. The statistical data presented in Figure 8 suggest that there is no significant difference between samples LL-37@AuNPs(2) and LL-37@AuNPs(4) and negative controls in terms of their ability to limit the growth of *Staphylococcus aureus*, *Escherichia coli*, *Bacillus cereus*, and *Candida albicans*. This indicates that, at a 10% concentration, samples LL-37@AuNPs(2) and LL-37@AuNPs(4) had the same inhibitory effect in preventing microbial growth. The existence of antimicrobial activity with a low inhibitory power indicates that peptides have antimicrobial active substances. However, in order to effectively suppress microbial development, the concentration must be increased. Comune et al. [10] found similar outcomes, noting that LL37 peptides (10 μg mL^−1^) or LL37-AuNPs (40 μg mL^−1^) did not exhibit significant antibacterial activity at lower concentrations. LL37-AuNPs, which are 80 μg mL^−1^, were reported to have strong antibacterial activity against both gram-positive and gram-negative bacteria. Notably, 80 μg mL−1 of LL37-AuNPs and 30 μg mL^−1^ of soluble LL37 peptide were needed to eradicate the bacteria. Furthermore, LL-37@AuNPs(2) and LL-37@AuNPs(4) samples exhibited a higher level of action in suppressing the development of *S. aureus* bacteria in contrast to *E. coli*, *B. cereus*, and *C. albicans* fungus. According to research conducted by Midorikawa et al. [23] and Cecotto et al. [12], antimicrobial peptide susceptibility is known to be important in *S. aureus* infection. These peptides may also have therapeutic applications.

### 3.7. Wound-Healing Activity

**Figure 9:**
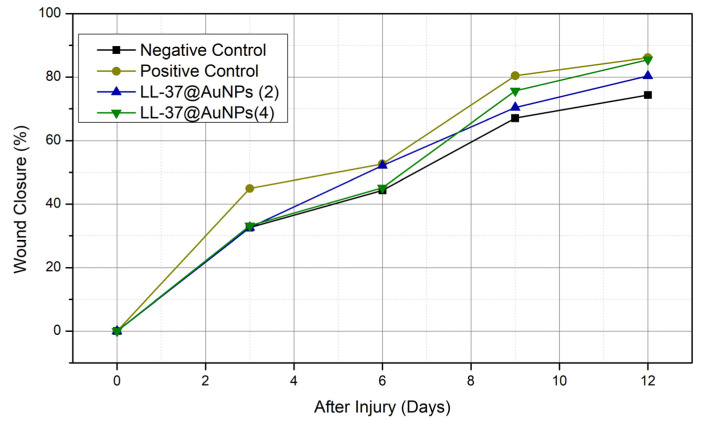
Wound closure % of sample LL-37@AuNPs(2) and LL-37@AuNPs(4) treatment on days 0, 3, 6, 9, and 12.

To assess the healing activity of the control group and the treatment groups (LL-37@AuNPs(2) and LL-37AuNPs(4), a wound excision was conducted. Table 1 and Figure 9 illustrate the LL-37@AuNPs(2) and LL-37@AuNPs(4) sample’s ability to close wounds.

Four exact and carefully programmed steps make up the typical biological process of wound healing. This process is mediated by complicated repair, which results in an inflammatory response and the production of reactive oxygen species (ROS), both of which are harmful. Furthermore, the infection caused by *P. aeruginosa* and *S. aureus* slows angiogenesis by delaying the inflammatory phase and interfering with the normal clotting mechanism. In this study, LL-37@AuNPs(2) and LL-37@AuNPs(4) were shown to have antibacterial activity in vitro. On the third day of treatment, the trial results showed no discernible differences between the treatment and control groups. Nonetheless, there are noticeable differences between the 9th and 12th days. There was not a statistically significant distinction in the average percentage of wound closure between the treatment and control groups. On the other hand, the process of wound healing is positively impacted by LL-37@AuNPs(2) and LL-37@AuNPs(4). 

In comparison to the negative controls, the wound-healing percentage rose to 85% on day 12 of the trial. LL-37@AuNPs(4) is the sample with the highest percentage of wound healing between days 3 and 12. It is noteworthy that, in comparison to sample LL-37@AuNPs(2), which has 0.22 µL of LL-37, this has 0.45 µL. As LL-37 was able to suppress the growth of *S. aureus* and manage the surrounding inflammation, it was suggested that the increased concentration of LL-37 contributed to the faster wound-healing rate in LL-37@AuNPs(4). According to Demirci et al. [13], LL-37 is one of the most well-characterized antimicrobial peptides and showed inhibitory effects on biofilm formation against *S. aureus*. In addition to their antimicrobial activity, these AMPs’ ability to prevent the production of biofilms makes them potentially valuable therapeutic agents [7]. Since LL-37 has a positive net charge, it may readily connect to the negatively charged cell membranes of bacteria, allowing it to interact with the cell membrane in order to reach its intracellular targets. Due to the opening creation in the cell membrane, this binding is assumed to be the primary mechanism causing the cellular integrity to deteriorate [13]. 

Furthermore, LL-37 affects multiple physiologically significant systems involved in wound healing. Inflammatory cells such as granulocytes and monocytes are drawn to peptides. It is established that LL-37 controls inflammatory sequences (cytokines) by releasing proteins and peptides during the inflammatory phase of wound healing [24]. LL-37 activates keratinocytes or cutaneous epithelial cells, and this stimulates growth factors in the outer layer of the skin by subsequent cell migration. Re-epithelialization and wound closure are thought to follow from this. It is pointed out by Tokumaru et al. [25] that the creation of new blood vessels following LL-37 treatment is attributed to the generation of vascular growth factors and the stimulation of endothelial cells in blood vessels. According to Xu et al. [14], treating *S. aureus* and *E. coli* with conjugated antimicrobial resistance with AuNPs caused the bacteria to disintegrate and breach their cell membranes, allowing the internal components to leak out. When *S. aureus*-infected mouse wounds were treated with laser radiation, the wounds healed more quickly, the inflammatory response was much reduced, and the damaged tissues were actively repaired.

The findings of this research open the door to a more thorough examination of this bioactive substance prior to its approval for use in therapeutic environments. Global research efforts have focused heavily on the quest for novel biomaterials with exceptional qualities for use in medicine. More recently, Chelu and Musuc [26] reported on a detailed examination into the evaluation of several multifunctional natural and synthetic biomaterials for advanced biomedical applications.

## 4. Conclusions

Using a simple one-step synthesis procedure, a nanomaterial based on LL-37@AuNPs conjugates was developed. The physico–chemical properties as well as the microstructure characters of LL-37@AuNPs conjugate, fulfil the requirements to be used as a bioactive material. It was discovered to be electrically stable and resistant to coagulation or aggregation. Due to its nanosized and other nanomaterial characteristics, the LL-37@AuNPs conjugate exhibits strong antibacterial activity and good resistance, particularly against *S. aureus* growth. These attributes make it a promising candidate for use as a drug-delivery platform. Even at low concentrations, LL-37@AuNPs conjugate still shows efficacy as an antibacterial and wound-healing substance. When LL-37 functions as an immunological regulator, it can also promote immune cell migration, which can help manage infection at high concentrations caused by bacteria or their byproducts. Additionally, by breaking down bacterial cell membranes and penetrating intracellular targets, this conjugate material exhibited exceptional effectiveness as a wound-healing agent. The results of this research pave the way for a more in-depth examination of the potential applications of metal nanoparticles and LL-37 antimicrobial peptides as accessible, affordable, and copious medicinal materials.

## Figures and Tables

**Table 1:** Morphology wound closure activity of sample LL-37@AuNPs(2) and LL-37@AuNPs(4) at days 0 and 12.

Sample	0 Days	12 Days
Negative Control	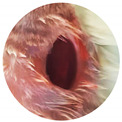	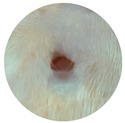
Positive Control	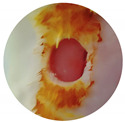	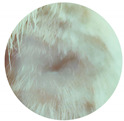
LL-37@AuNPs(2)	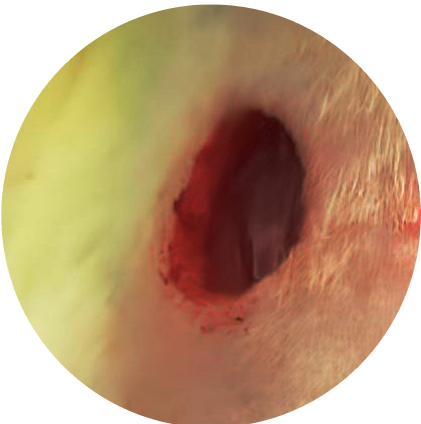	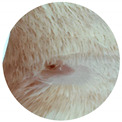
LL-37@AuNPs(4)	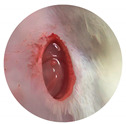	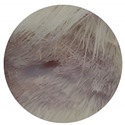

## Data Availability

Data are contained within the article.
